# Fibrinogen Concentrate in Acute Hemorrhage: Mechanistic Insight, Thresholds, and Targeted Replacement

**DOI:** 10.3390/jcm15166156

**Published:** 2026-08-07

**Authors:** Niels Rahe-Meyer, Justyna Bartoszko, Jerrold H. Levy

**Affiliations:** 1 Clinic for Anesthesiology and Intensive Care Medicine, Franziskus Hospital, 33615 Bielefeld, Germany; 2 Department of Anesthesiology and Pain Medicine, University of Toronto, Toronto, ON M5G 1E2, Canada; 3 Department of Anesthesia and Pain Management, Toronto General Hospital-University Health Network, Toronto, ON M5G 2C4, Canada; 4 Department of Anesthesiology, Critical Care Medicine, and Surgery (Cardiothoracic), Duke University School of Medicine, Durham, NC 27710, USA; 5 The Centre for Blood Research, University of British Columbia, Vancouver, BC V6T 1Z3, Canada

**Keywords:** acquired fibrinogen deficiency, hypofibrinogenemia, hemorrhage, surgery, coagulopathy

## Abstract

Fibrinogen is an essential component of hemostasis and clot formation that stabilizes the platelet-dependent primary hemostatic process. Low fibrinogen levels can be primary (congenital) or secondary (acquired). Acquired hypofibrinogenemia may result from chronic diseases (e.g., liver, autoimmune diseases, and malignancies) or major hemorrhage (e.g., trauma, surgery). Low fibrinogen levels are both a symptom and a precipitating factor for coagulopathy and ongoing bleeding. Fibrinogen repletion with fibrinogen-containing products is important for managing coagulopathic bleeding with suspected or documented hypofibrinogenemia. Different available fibrinogen sources include fibrinogen concentrate, cryoprecipitate, and frozen plasma and vary based on multiple factors including fibrinogen content, purity, other clotting or non-clotting proteins (e.g., immunomodulating proteins or proteins of unknown function), preparation time, safety, volumes, and availability. Fibrinogen replacement strategies have been studied in trauma but also in patients undergoing spine, cytoreductive, and cardiac surgery. In this review, we discuss the physiological actions of fibrinogen, strategies for control of coagulopathic bleeding related to hypofibrinogenemia, and the therapeutic, logistical, and economic factors that influence treatment decisions. In addition, current guidelines and clinical studies were considered regarding the formation of evidence-based treatment strategies that can be individualized at the patient’s bedside.

## 1. Introduction

Fibrinogen is a critical factor in clot formation and the maintenance of hemostasis [1]. In this review, we will provide background on the physiological effects of fibrinogen and discuss the clinical significance of low fibrinogen levels in surgical and trauma settings, with a particular focus on cardiac surgery. Strategies for optimal bleeding management are addressed in the light of treatment availability and recent clinical evidence. Current strategies and guidelines for the use of fibrinogen concentrates, including how they can be applied to support evidence-based bedside decisions, will also be considered.

## 2. The Role of Fibrinogen in Clot Formation and Hemostasis

The equilibrium between coagulation and fibrinolysis is essential for effective hemostasis. Central to this process is fibrinogen, the most abundant circulating coagulation protein, which serves not only as the precursor of fibrin but also as a key determinant of clot structure and mechanical stability [2,3]. Following vascular injury, thrombin cleaves fibrinopeptides A and B from fibrinogen, generating fibrin monomers that spontaneously polymerize into oligomers and subsequently assemble into protofibrils. These protofibrils laterally aggregate, branch, and are further stabilized through factor XIII-mediated cross-linking to form a three-dimensional fibrin network that reinforces the initial platelet plug generated during primary hemostasis [3,4]. Factor XIII-mediated cross-linking increases fiber stiffness and clot elasticity, reduces clot permeability, and enhances resistance to plasmin-mediated fibrinolysis, thereby improving clot durability under conditions of ongoing hemorrhage [3,4,5].

Fibrinogen binds activated platelets through the glycoprotein IIb/IIIa (GPIIb/IIIa) receptor, one of the most abundant platelet surface receptors, representing up to 15% of total platelet surface proteins [6,7,8,9]. This interaction mediates platelet aggregation while strengthening platelet–fibrin interactions that promote clot contraction and further stabilize the developing thrombus [3]. Fibrinogen concentration is a major determinant of fibrin network architecture and, consequently, clot function. Fibrin network density, fiber diameter, branching, and pore size govern clot elasticity, viscoelastic strength, permeability, and susceptibility to fibrinolysis [3,4,5,10]. Higher fibrinogen concentrations promote the formation of dense, highly branched fibrin networks with smaller pores and greater mechanical strength, producing clots that are more resistant to mechanical disruption and plasmin-mediated degradation (premature fibrinolysis) [3,4,5,10]. Additionally, fibrinogen binds to other cell types to foster wound healing and tissue regeneration and helps to prevent infection, both through its actions as a physical barrier as well as its ability to recruit immune cells [2,11,12].

Fibrinogen deficiencies can be congenital or acquired. Acquired fibrinogen deficiencies can arise from numerous disease states and events including liver disease, autoimmune diseases, malignancies, trauma, obstetric complications, or surgery [2,13,14]. Patients are generally considered at risk for increased severity of coagulopathic bleeding when fibrinogen levels are below 200 mg/dL, although this may vary by specific patient population [15,16,17]. Because fibrinogen is frequently the first coagulation factor to decline to critically low levels during major hemorrhage, early replacement has a strong biological rationale.

It is thought that restoring fibrinogen not only replenishes substrate for fibrin polymerization but also re-establishes fibrin network architecture, clot mechanical strength, and resistance to fibrinolysis [3,4,5,10,16]. Fibrinogen levels are an important factor in determining the structure of fibrin clots. Low fibrinogen levels result in a less compact clot structure with thicker fibers [18,19,20]. This less compact clot structure has been associated with greater permeability and easier access for fibrinolytic agents [18,19,21]. Decreased clot density and increases in permeability, fiber diameter, and fibrinolysis are all associated with bleeding complications [22].

Acquired fibrinogen deficiency resulting from trauma and major surgical bleeding will be the focus of this review. Acquired fibrinogen deficiency in the context of postpartum hemorrhage was not addressed in this review. The evidence from controlled clinical trials on fibrinogen replacement in postpartum hemorrhage is extremely limited and suggests that in the multifactorial nature of this condition, fibrinogen replacement therapy alone may not be effective [23,24,25].

## 3. Fibrinogen Replacement Therapies

When fibrinogen deficiency is identified in the context of a severe bleeding event, there are three primary treatment options. All are derived from human plasma. Frozen plasma (FFP), cryoprecipitate (Cryo), and fibrinogen concentrate (FC) have historically been used to replenish fibrinogen levels in acquired hypofibrinogenemia with clinically significant bleeding (Table 1) [13,26].

### 3.1. Frozen Plasma

Plasma was first introduced as a therapeutic blood product in a dried (lyophilized) form in the 1940s, with citrated whole blood being the other major option for fibrinogen replacement [27]. Frozen plasma (FP) became more widespread once technologies and materials (such as polyvinyl chloride collection bags) became available to separate whole blood more readily into individual components [28]. During this time, plasma was an inefficient source of exogenous clotting factors and fibrinogen replacement due to its high variability and overall low concentration (1.6–5 g/L; 160–500 mg/dL) [29], requiring high volumes of plasma to treat coagulopathic bleeding. However, especially for fibrinogen repletion, isovolumic replacement will not increase fibrinogen to critical levels needed for clot formation. A typical pediatric and adult dose of plasma is approximately 15 mL/kg, and replenishing a given patient’s plasma fibrinogen level above 1.5 g/L (150 mg/dL) may require administering an extremely large volume. In such cases, 15 mL/kg or even 30 mL/kg may be insufficient. This can lead to adverse events and circulatory overload [29].

FP contains all clotting factors and other plasma proteins, including anticoagulant proteins such as Proteins C, S, and antithrombin [30,31]. The presence of all clotting factors could be of benefit in hemostasis but also increase thrombogenic risk [32,33]. However, the presence of other plasma proteins and microparticles could increase the risk of alloimmunization and hypersensitivity reactions and transfusion-related acute lung injury (TRALI) [34,35,36]. TRALI is neutrophil-mediated damage to the pulmonary vasculature caused by antibodies in donor blood reacting with antigens in the recipient. Damage to the vascular endothelium leads to leakage of proteins and fluid into the alveolar space [36]. Another potential disadvantage of FP is the lack of viral inactivation unless the plasma is from a commercial source. As noted, fibrinogen content in FP is variable, and, compared to Cryo and FC, larger volumes of FP are required, which cannot consistently increase fibrinogen levels [1,16]. These larger volumes are also associated with a greater risk of transfusion-associated circulatory overload (TACO) [37], and its use is associated with longer hospital stays, increased costs [38], and increased morbidity and mortality associated with transfusions [39].

### 3.2. Cryoprecipitate

Cryoprecipitate was introduced in the 1960s after the discovery of a method of thawing plasma that allowed “cold insoluble” components to precipitate. This byproduct was found to be a rich source of fibrinogen and factor VIII, and it initially gained traction as a treatment option for hemophilia A and congenital fibrinogen disorders. In the US, the American Red Cross specifies that each single unit of cryoprecipitate contains approximately 700 mg of fibrinogen in a 24 mL volume, and each pooled unit of cryoprecipitate contains 3 g of fibrinogen per 100 mL [40]. The Association for the Advancement of Blood & Biotherapeutics (AABB) states that every unit of Cryo should contain ≥150 mg of fibrinogen in 5 to 20 mL [41]. In the UK, a typical bag of pooled cryoprecipitate is prepared from five single donations and contains >700 mg of fibrinogen (mean 1596 mg) and >350 IU of factor VIII (mean of 499 IU) at a typical volume of 100–300 mL [42].

Due to its small volume and storage capacity of up to 1 year at −18 °C, its use expanded rapidly for the treatment of acquired fibrinogen deficiency [43]. However, it requires thawing for subsequent use, and, once thawed, it is only usable for up to 4 h. Importantly, although cryoprecipitate is a concentrated source of fibrinogen and factor VIII, it also contains von Willebrand factor (vWF), fibronectin, factor XIII, and platelet microparticles—with some of these constituents having an unclear association with thromboembolic complications (Table 2) [44,45,46,47].

For many years, cryoprecipitate was the primary treatment for fibrinogen deficiency in many countries. However, in some countries where fibrinogen concentrate was available, cryoprecipitate was already removed from use for safety reasons [13,16,26]. Cryo does not undergo all the procedures for virus removal or inactivation used in the manufacture of FCs and may pose a greater risk of viral transmission, especially as a multidonor product [48]. In addition, due to variable fibrinogen content in donor plasma, Cryo does not have a standardized or predictable amount of fibrinogen [16]. In contrast to FCs, testing for ABO blood type compatibility is typically recommended for Cryo, thawing of the frozen product is required, and, as previously noted, the product must be administered within 4 h.

**Table 2:** Coagulation protein content and concentration in cryoprecipitate (Cryo), fresh frozen plasma (FFP), and fibrinogen concentrate (FC).

Coagulation Protein	Cryo Content per Bag or Concentration	FFP Content per Bag or Concentration	FC Content per Vial or Concentration
Content			
Fibrinogen	183 ± 44 mg [49]	725 ± 199 mg [50]	900–1300 mg [50]
Factor VIII	133 ± 37 IU [49]	265 ± 83 IU [49]	
Factor XIII	60 ± 30 IU [49]	288 ± 77 IU [49] 1.6–3.1 mg [49]	
von Willebrand Factor	168 ± 34 IU [49]	221 ± 65 IU [49] 2.5–4 mg [51]	
Concentration			
Fibrinogen	4.83 ± 1.43 g/L [42] 483 ± 143 mg/dL	1–3 g/L [50] 100–300 mg/dL	19.73 ± 1.22 g/L [42] 1973 ± 122 mg/dL
Factor VIII	210.03 ± 135.01 IU/dL [42]	>70 IU/mL [52]	<10 IU/dL [42]
Factor XIII	192.17 ± 62.70 IU/dL [42]		328.33 ± 20.41 IU/dL [42]
von Willebrand Factor	212.75 ± 37.12 IU/dL [42]		17.00 ± 2.61 IU/dL [42]
Fibronectin	168.97 ± 94.99 µg/mL [42]	55 mg/bag [53]	15.58 ± 3.11 µg/mL [42]

### 3.3. Fibrinogen Concentrate (FC)

Like cryoprecipitate, pathogen-inactivated and purified fibrinogen concentrates (FCs) are isolated from human plasma. This treatment option includes viral inactivation during manufacturing and the removal of procoagulation factors and immunomodulatory proteins but provides a standardized dose for repletion in a small administration volume. Further, FCs can be prepared for administration more rapidly than Cryo and FFP [26,54]. Each 1 g of fibrinogen concentrate is reconstituted in approximately 50 mL of sterile water (20 mg/mL). They contain a reliable, standardized amount of fibrinogen, are easier to prepare (as they lyophilized and rapidly reconstituted), and are easy to store (some can be kept in original packaging at room temperature) [55,56,57]. Fibrinogen concentrates have been available in some European countries for decades; however, they are now gaining popularity in other jurisdictions for acquired hypofibrinogenemia. The properties of the three available treatments are shown in Table 1.

## 4. Clinical Studies Comparing Fibrinogen Replacement Therapies

Multiple studies have assessed the efficacy and safety of Cryo, FP, and FC in bleeding management after trauma and perioperatively for surgical patients. The studies described below were either pilot studies addressing feasibility of planned larger studies on this topic or studies designed to assess therapeutic equivalence or a potential advantage of FC over the other treatments.

### 4.1. Trauma

For trauma, three relatively small feasibility pilot studies of FC have been conducted. They are the FiiRST pilot study [58], the E-FIT pilot study [59], and the FEISTY pilot study [60] (Table 3). In the FiiRST study, 50 hypotensive (systolic blood pressure ≤100 mm Hg) adult trauma patients requiring a blood transfusion were randomly assigned to receive either 6 g of FC or placebo (saline) administered in a double-blind manner. The primary endpoint of the study was to determine the feasibility of administering FC within 60 min of hospital arrival. Overall, 96% of patients received their assigned treatment within 60 min (95% FC; 96% placebo). Not surprisingly, fibrinogen levels were higher in the FC group for up to 12 h after admission, with the largest difference observed at the 3 h mark post-administration (2.9 mg dL^–1^ vs. 1.8 mg dL^–1^; *p* < 0.01). There were no differences between the groups in 28-day mortality or thromboembolic events; however, the study was not powered to detect a difference [58].

The E-FIT 1 study was also a pilot randomized, placebo-controlled trial aimed at determining the feasibility of administering FC within a short time after hospital admission. The target was for 90% of patients to receive their assigned treatment within 45 min. In the E-FIT1 trial, adult trauma patients with active bleeding that qualified as major hemorrhage were enrolled. A total of 27 of the 39 participants in the study (69%) received their assigned treatment within 45 min of admission. At 2 h after admission, fibrinogen levels were significantly higher in the FC group (2.8 ± 1.3 g/L (280 ± 130 mg/dL); mean ± SD) than in the placebo group (1.8 ± 0.6 g/L (180 ± 60 mg/dL); *p* < 0.0001). There were no indications of differences in adverse events or mortality between the treatment groups, although, as a pilot study, it was not designed to detect such differences [59].

The objective of the FEISTY study was to compare the time to fibrinogen replacement in severely injured patients with major hemorrhage and hypofibrinogenemia using FC and Cryo as part of a multicenter pilot study. Of the 100 patients randomized for the study, 62 were hypofibrinogenemic and eligible for inclusion (FC *n* = 37; Cryo *n* = 25). Dosing was guided by a FIBTEM A5 strategy. The mean time to infusion was shorter for FC (median 29 min; interquartile range (IQR) 23–40 min) than for Cryo (median 60 min; IQR 40–80 min; *p* = 0.0001). Fibrinogen levels before and after treatment, adverse events, and mortality were similar between the groups; however, as a pilot study, it was not powered to definitively establish differences [60].

A retrospective study of patients receiving fibrinogen supplementation for traumatic hemorrhage evaluated 85 patients who received FC and 170 patients that received Cryo [61]. While there were no differences in major complications or mortality, patients receiving FC required fewer units of blood products (packed red blood cells (PRBCs), FFP, and platelets) and had shorter intensive care and hospital stays than the patients that received Cryo.

**Table 3:** Placebo-controlled and comparator trials with fibrinogen concentrate (FC) trauma patients and patients undergoing cytoreductive, spinal, or cardiac surgery.

Study	Pts Treated with FC (*n*)	Pts Treated with Comparator (*n*)	Pts Analyzed (*n*)	Primary Endpoint	Efficacy	Safety
**Trauma**						
FiiRST Pilot [58]	6 g FC (21)	Placebo (24)	(44)	Feasibility of delivery within 60 min	96% Overall (95% FC, 96% placebo) Fibrinogen levels higher with FC up to 12 h	Comparable
E-FIT Pilot [59]	6 g FC (24)	Placebo (14)	(28)	Feasibility of delivery to 90% of pts within 45 min	69% Overall (range 10–82 min) Fibrinogen levels higher in FC group at 2 h	Comparable
FEISTY Pilot [60]	3 g FC (37) Based on FIBTEM A10	8 U Cryo (25)	(62)	Feasibility–Time from ROTEM blood draw to administration	Faster for FC (29 min) vs. Cryo (60 min) Fibrinogen levels and use of allogeneic blood products similar	Comparable
**Cytoreductive or Spinal Surgeries**						
FORMA-05 [44] Cytoreductive/ PMP	4 g FC (21) Based on FIBTEM A20	10 U Cryo (22)	(43)	Composite of intra- and post-op hemostatic efficacy	FC non-inferior FC arrived 46 min sooner Fibrinogen levels higher in FC group and better FIBTEM A10	Comparable
AdFirst [45] Major spinal or cytoreductive/ PMP	2–8 g FC (103) Based on bleeding and FIBTEM A10	FFP 15 mg/kg or 10 U Cryo (98)	(201)	Intraoperative blood loss from decision to treat until the end of surgery	FC non-inferior Significantly less blood loss in FC group (279 mL)	Comparable but numerically lower in FC group Significantly less thromboembolic events in FC group
**Cardiac Surgeries**						
ZEPLAST [62]	4 g (IQR 3 to 6) Based on FIBTEM MCF (58)	Placebo (58)	(116)	Allogeneic blood products used during hospital stay	More FC patients free from use of allogeneic blood products	Comparable
REPLACE [63]	6 g FC (78)	Placebo (74)	(142)	Allogeneic blood products 24 h post-administration	FC inferior 2013 pilot study had opposite results [64]	Comparable
FIBRES [65]	4 g FC (372) Based on FIBTEM A10	10 U Cryo (363)	(735)	Allogeneic blood products 24 h post-administration	FC non-inferior overall Superior in elective surgery	Comparable

PMP = pseudomyxoma peritonei; FIBTEM = ROTEM without the contribution of platelets with inhibition of platelet activity by cytochalasin D; ROTEM = rotational thromboelastometry; Cryo = cryoprecipitate; A10, A20 = ROTEM clot firmness amplitude at 10 min and 20 min; MCF = ROTEM maximum clot firmness; IQR = interquartile range.

### 4.2. Cytoreductive or Spinal Surgeries

Table 3 shows the results of two comparator studies for FC in patients undergoing cytoreductive or spinal surgeries. In the FORMA-05 study, the comparator was Cryo [44], and in the AdFIrst study, the comparator was either FFP or Cryo [45].

The type of surgery studied in the FORMA-05 study was cytoreductive surgery with hyperthermic intraperitoneal chemotherapy for pseudomyxoma peritonei (PMP). PMP usually arises after perforation of a primary tumor in the appendix, leading to the dissemination of mucus and tumor cells throughout the abdominal cavity [66,67]. Cytoreductive surgery for PMP is often lengthy, complex, and associated with extensive blood loss [68,69,70] and acquired hypofibrinogenemia [44].

In the FORMA-05 study, patients undergoing cytoreductive surgery for PMP with predicted blood loss ≥ 2 L were treated with 4 g FC or 2 × 5 U Cryo. The primary endpoint was successful hemostasis during and after surgery. The endpoint was a composite of intraoperative and post-operative (24 h) ratings of hemostatic efficacy on a four-point scale: (excellent, good, moderate, or none). Interoperative hemostasis was assessed by both the surgeon and anesthesiologist, and 24 h post-operative hemostasis was assessed by a hematologist.

All patients in both treatment groups achieved hemostasis. FC was non-inferior to Cryo and was available for administration in the operating room on average 46 min earlier. Increases in fibrinogen levels were higher with FC (0.78 g/L (78 mg/dL)) than with Cryo (0.35 g/L (35 mg/dL); *p* < 0.0001). For adverse events, the treatments were similar except for thromboembolic events, which were observed only in the Cryo group [44].

The AdFIrst study compared FC with FFP or Cryo in 201 patients undergoing cytoreductive surgery for PMP or major spinal surgery. FC (2–8 g) was administered to 103 patients and FFP (15 mL/kg) or Cryo (10 U) was administered to 98 patients. PMP surgery patients were randomized if blood loss was expected to be >2 L during surgery, and spinal surgery patients were randomized if blood loss was >1 L. Laboratory values were not used to trigger treatment, but dose was calculated based on FIBTEM A10, targeting a return to each patient’s baseline level. Patients undergoing PMP surgery received either 4 g FC or 10 U Cryo, whereas patients undergoing spinal surgery received 2–8 g FC or 15 mL/kg FFP. The primary endpoint was intraoperative blood loss during surgery from the treatment decision to the end. Overall intraoperative blood loss was less (and non-inferior) in the FC group than in the FFP/Cryo group (−279 mL least squares mean difference; *p* < 0.0001) and in the spinal (*p* = 0.00075) and abdominal subgroups (*p* = 0.011). Post hoc superiority analysis showed that FC was superior to FFP overall (*p* = 0.0087) and in the spinal surgery subgroup (*p* = 0.013) but not in the abdominal surgery subgroup (*p* = 0.15) (Table 4) [45].

Secondary endpoints showed that more patients in the FC group had successful correction of their fibrinogen levels at ≤15 min after administration (*p* < 0.0001) and fewer patients had post-operative bleeding (up to day 8 post-surgery) in the FC group (0) than in the FFP/Cryo group (*p* = 0.028). There were no significant differences in the other secondary endpoints (Table 4).

### 4.3. Cardiac Surgeries

**Figure 1:**
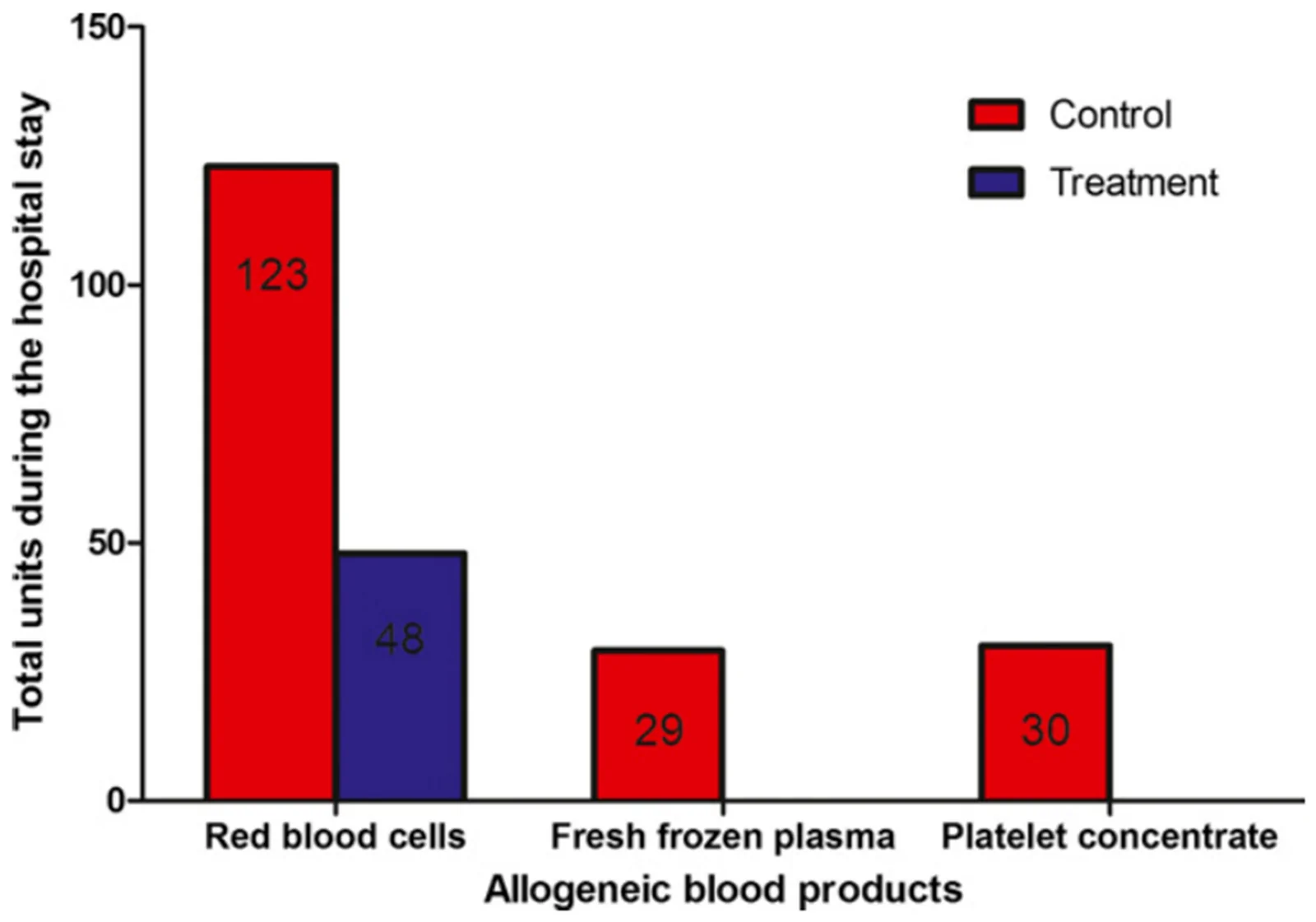
The amount of allogeneic blood products transfused post-operatively in cardiac surgery patients (from the end of cardiopulmonary bypass to discharge from the hospital). Control = placebo (saline administration); Treatment = fibrinogen concentrate (from Ranucci et al. [62] published open access under a CC-BY-NC license).

For cardiac surgery, three important controlled trials with FC were ZEPLAST [62], REPLACE [63], and FIBRES [65]. ZEPLAST was a double-blind, placebo-controlled study of FC conducted in 116 patients undergoing heart surgery with CPB expected to last longer than 90 min [62]. Patients in the treatment arm received FC after protamine administration. Patients in the control arm received normal saline. The primary endpoint was reduction in post-operative allogeneic blood product use, reflecting post-operative bleeding. Figure 1 shows that administration of FC significantly reduced the use of allogeneic blood products (odds ratio 0.40, 95% confidence interval 0.19 to 0.84, *p* = 0.015). Post-operative bleeding was also reduced: median 300 mL in the treatment arm (interquartile range (IQR) 200 to 400 mL) compared to 355 mL (IQR 250 to 600 mL); *p* = 0.042 [62].

The REPLACE study compared FC with placebo, and the FIBRES study compared FC with Cryo (Table 3). The REPLACE study included 142 patients undergoing aortic surgery (thoracic or thoracoabdominal) with CPB. FC was dosed based on the FIBTEM MCF (target 22 mm). Placebo or FC was given if the bleeding mass 5 min after protamine was 60–250 g. If mass was >250 g, patients were excluded from the study. A treatment algorithm was used to determine the administration of platelets and FFP in these patients [63].

In the REPLACE study, the reduction in bleeding mass between pre-treatment and post-treatment measurements was similar for the FC group (20 g) and the placebo group (19.5 g; *p* = 0.32), the use of allogeneic blood products was higher in the FC group (5.0 U) than in the placebo group (3.0 U; *p* = 0.026), and the percentage of patients avoiding transfusion was lower in the FC group (15.4%) than in the placebo group (28.4%; *p* = 0.047) [63].

The results of the 2016 REPLACE study differed from those of the 2013 pilot study on which it was based [64]. In the pilot study, 61 patients were randomized (29 received FC; 32 received placebo), and the FC group received fewer units of allogeneic blood products (2 U) compared to placebo (13 U; *p* < 0.001). Total avoidance of transfusions was higher in the FC group (100%) than in the placebo group (45%; *p* < 0.001) [64].

The authors attributed these differences to low bleeding rates and high fibrinogen levels overall in the REPLACE study. They also cited variability in adherence to the transfusion algorithm and the possibility that the 5 min bleeding mass was not an ideal inclusion criterion for the study [63]. The study was also conducted at a relatively large number of sites with small patient numbers at each site, which may have compounded differences in transfusion practices.

**Figure 2:**
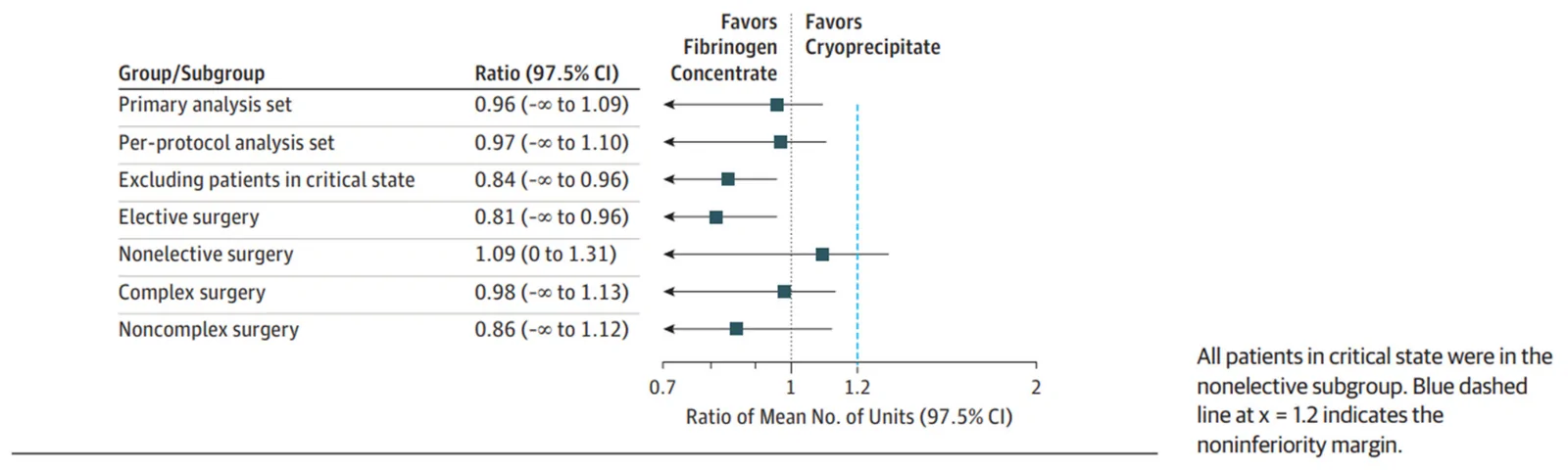
Ratio of mean number of allogeneic blood products transfused after cardiopulmonary bypass. Squares indicate the ratios and the lines indicated the 97.5% confidence intervals. The arrows indicate values that lie beyond the limits of the figure. The blue dotted line at a ratio of 1.2 is the cutoff for FC non-inferiority and the black dotted line at 1.0 is the cutoff for FC superiority (Reproduced with permission from Callum et al. doi:10.1001/jama.2019.17312. Copyright © 2019 American Medical Association, All rights reserved, including those for text and data mining, AI training and similar technologies [65]).

The FIBRES trial was a randomized controlled trial with a 1:1 allocation of patients to treatment with FC or Cryo. This was a non-inferiority trial comparing FC to Cryo. All patients had CPB and were randomized when they had significant post-bypass bleeding determined to be the result of hypofibrinogenemia (plasma level < 2.0 g/L (200 mg/dL) by the Clauss method or by FIBTEM). The study was conducted at 11 sites in Canada. The patient population analyzed was 735, with 372 receiving FC (4 g) and 363 receiving Cryo (10 U). The mean difference in allogeneic blood products used was −0.73 (FC 16.3 U versus Cryo 17.0 U), and FC was found to be non-inferior to Cryo (ratio 0.96; *p* < 0.001) (Figure 2) [65].

The baseline characteristics of the two treatment groups in the FIBRES trial were similar, except for the presence of critical illness before surgery. Critical illness was determined by blinded adjudication on patients who underwent emergency surgery and had any of the following conditions: (1) ventricular tachycardia or fibrillation or cardiac arrest; (2) pre-operative cardiac massage; (3) pre-operative ventilation before anesthetic room; (4) hemodynamic support requiring pre-operative inotropes or ventricular assist devices; (5) pre-operative acute renal failure (anuria or oliguria); (6) acute aortic dissection. The percentage of these patients was higher in the FC group (17%) than in the Cryo group (11%). Subgroup analysis showed non-inferiority in this subgroup and in all other subgroups (elective surgery, complex surgery, and non-complex surgery) except for non-elective surgery (Figure 2). FC showed superiority when patients in a critical state were excluded and when patients undergoing elective surgery were included [65].

In addition to these larger studies, some smaller studies have been conducted comparing FC to Cryo in cardiac surgery patients. A randomized, controlled study in infants (<12 months) showed that patients who received FC (*n* = 29) received fewer units of allogeneic blood products than patients who received Cryo (*n* = 2 9; *p* = 0.003) [71]. Another study in children (<7 years) found there was no difference between treatment with FC (*n* = 30) and Cryo (*n* = 33) in the number of units of allogeneic blood products administered and in outcomes [72]. A single-center randomized study in adults found that Cryo (*n* = 40) and FC (*n* = 48) were both effective in increasing plasma fibrinogen levels with no substantial differences in outcomes or adverse events [73]. A meta-analysis of these studies plus the FIBRES study [65] found no significant differences between FC and Cryo in terms of mortality, use of allogeneic blood products, or post-op thrombosis or blood loss [74].

### 4.4. Overall Safety

Although fibrinogen concentrate enhances clot formation, current evidence does not demonstrate a clinically significant increase in thromboembolic events following its use for acquired bleeding. Randomized trials in cardiac surgery and other perioperative settings have reported similar or lower rates of venous and arterial thromboembolic events with fibrinogen concentrate compared with cryoprecipitate or FP [45,65,75,76], findings that are supported by systematic reviews and meta-analyses [75,76]. Post-marketing pharmacovigilance data encompassing more than 30 years of clinical use and approximately 7.5 million grams of fibrinogen concentrate administered worldwide have similarly demonstrated a low incidence of thromboembolic adverse drug reactions [77,78]. In contrast, retrospective cohort studies have reported an association between cryoprecipitate transfusion and thromboembolic complications, although these findings are susceptible to confounding by indication [79]. Collectively, the available evidence supports a favorable safety profile for fibrinogen concentrate when used appropriately.

## 5. Association of Fibrinogen Levels with Bleeding and Replacement Thresholds

A universal fibrinogen threshold for replacement may not be appropriate across all clinical settings, as the mechanisms and circumstances underlying acquired hypofibrinogenemia may differ substantially. In trauma and postpartum hemorrhage, fibrinogen depletion often occurs early as a result of acute consumption, hemorrhage, dilution, and hyperfibrinolysis [80,81,82]. In contrast, bleeding in cardiac surgery shares many of these concerns but tends to be more multifactorial and also reflects cardiopulmonary bypass-induced platelet dysfunction, coagulation factor consumption, and inflammatory activation. Lastly, bleeding in liver transplantation is often related to portal and central venous pressure, with patients that may have decreased fibrinogen levels but a dynamic rebalanced hemostatic state [80,81,83,84]. Consequently, the optimal fibrinogen concentration required to maintain adequate hemostasis is likely to vary significantly, and differences in contexts and patient populations should be considered [81,82,85,86,87].

### 5.1. Measurement of Fibrinogen Levels

Current guidelines recommend fibrinogen levels be monitored by the Clauss assay or a point-of-care method, usually viscoelastic testing during major bleeding after trauma [80] or during severe perioperative bleeding [84]. The three main assays for measuring fibrinogen levels are summarized in the table below (Table 5).

The Clauss method is the most commonly used method for assessing fibrinogen levels. This approach measures coagulation time in citrate plasma containing excess thrombin [88,89,90]. The protocol for this assay and the reagents used can differ between laboratories, and results can be altered by the ratio of low to high molecular weight fibrinogen and the presence of fibrin or fibrinogen degradation products [89,91]. Additionally, the turnaround times of the Clauss assay can be slow and can vary from site to site, but one study reported a range of 50–100 min [92], and results can be affected by high heparin concentrations and direct thrombin inhibitors [93,94,95,96,97,98,99].

Two of the most common viscoelastic testing systems are rotational thromboelastometry (ROTEM) and thromboelastography (TEG) [88]. These methods detect mechanical resistance of a rotating pin placed in the blood sample. The results from ROTEM and TEG can be returned in as little as 5–10 min [100]. Coagulation for ROTEM is initiated by either tissue factor (EXTEM) or ellagic acid (INTEM). Fibrinogen function in ROTEM is isolated when the contribution of platelets is removed by inhibition of platelet activity by cytochalasin D or a combination of cytochalasin D and abciximab (FIBTEM). Clotting time (CT: the lag time before clot formation), clot formation time (CFT), α, the angle which reflects the initial rate of clot formation, firmness amplitude at 10 min (A10), and maximum clot firmness (MCF) were assessed.

**Table 5:** Summary of common functional fibrinogen assays.

Assay	Potential Advantages	Potential Disadvantages
Clauss	“Gold Standard” [14,101] Robust to low levels and heparin up to ~3 U/mL [102] Clinical relevance to guidelines [14]	Slow turnaround time (30–90 min) [101,103] Can be inaccurate in trauma (dysfibrinogenemia or altered fibrin polymerization) [104] Cannot determine quantitative vs. qualitative deficits [105,106] Interference by direct thrombin inhibitors [93,94,95,96,97,98,99]
Rotational Thromboelastometry (ROTEM)	Rapid POC results (10–20 min) [101] Strong correlation to Clauss in several groups [103,107,108] Good accuracy at low levels [109,110] Correlation to Clauss better than TEG in some comparative studies [110,111]	Correlation with Clauss degrades in some circumstances [112,113,114] Device differences and non-comparability [115] Reflects fibrin function—can be low when Clauss is normal [116]
Thromboelastography (TEG)	Rapid POC results [101] Good to strong correlation with Clauss in many populations [101,117,118,119] Good results at low fibrinogen in certain settings [118] Cartridge-based assays simplify use [120,121]	Can overestimate levels compared to Clauss [110,122] Accuracy decreases with trauma, ESRD, and extreme levels [123]

A recent study by Hartmann et al. examined the sensitivity and selectivity of a TEG multichannel cartridge hemostasis analyzer that includes a citrated functional fibrinogen assay with heparin neutralization capacity [124]. This study found consistently high sensitivity and robust sensitivity for this assay and close correlation with the Clauss method. They identified a cutoff maximum amplitude of 19.5 mm corresponding to 200 mg/dL (2.0 g/L) with a sensitivity of 86% and a specificity of 78%.

Two meta-analyses were conducted on controlled clinical trials using viscoelastic assays to guide hemostatic therapy compared with standard laboratory tests. One analysis examined only data from perioperative studies. This study found that the use of viscoelastic testing produced significant reductions in mortality (relative risk (RR) = 0.64; *p* = 0.03) and acute kidney injury (RR = 0.53; *p* = 0.005). In addition, the risks of platelet and FFP infusions were reduced along with the volume of RBCs and FFP. No differences were seen in thrombotic events, length of hospitalization, or length of ICU stay [125]. The other meta-analysis included other patient types (i.e., liver transplant recipients and burn patients), but, despite its broader inclusion criteria, 96% of the patients included were cardiac surgery patients. Their analysis showed a significant reduction in mortality and in the percentage of patients receiving pRBCs, FFP and platelets, but, in all cases, they rated the quality of evidence as low due to the infrequency of the measured events and poor trial design [126].

These technologies all have their limitations. The Clauss assay, although considered the gold standard [14,101], has a slower turnaround time than point-of-care tests [101,103], but this can vary greatly between institutions and can potentially be shortened for emergencies. When initially introduced, there was considerable variability in results within and between institutions in the results of viscoelastic testing [127,128]. In addition, it should be noted that values from one type of viscoelastic testing (e.g., ROTEM) cannot be directly extrapolated to another type (e.g., TEG) [129,130,131].

A newer method for point of care coagulation testing is sonorheometry, which utilizes sonic estimation of elasticity via resonance (SEER) technology. High-frequency ultrasound is used to generate a shear wave which causes the sample to resonate [132]. Studies have been conducted comparing sonorheometry with traditional laboratory testing (e.g., the Clauss assay), and some have found a strong correlation [117,133,134]. Other studies have compared sonorheometry with other point-of-care hemostasis analyzers (ROTEM and TEG) and have found a strong correlation but warn that the values are not interchangeable [135,136,137,138]. Additional large-scale studies will likely be necessary for incorporation of this technology into treatment algorithms and guidelines.

### 5.2. Hypofibrinogenemia in Cardiac Surgery

**Figure 3:**
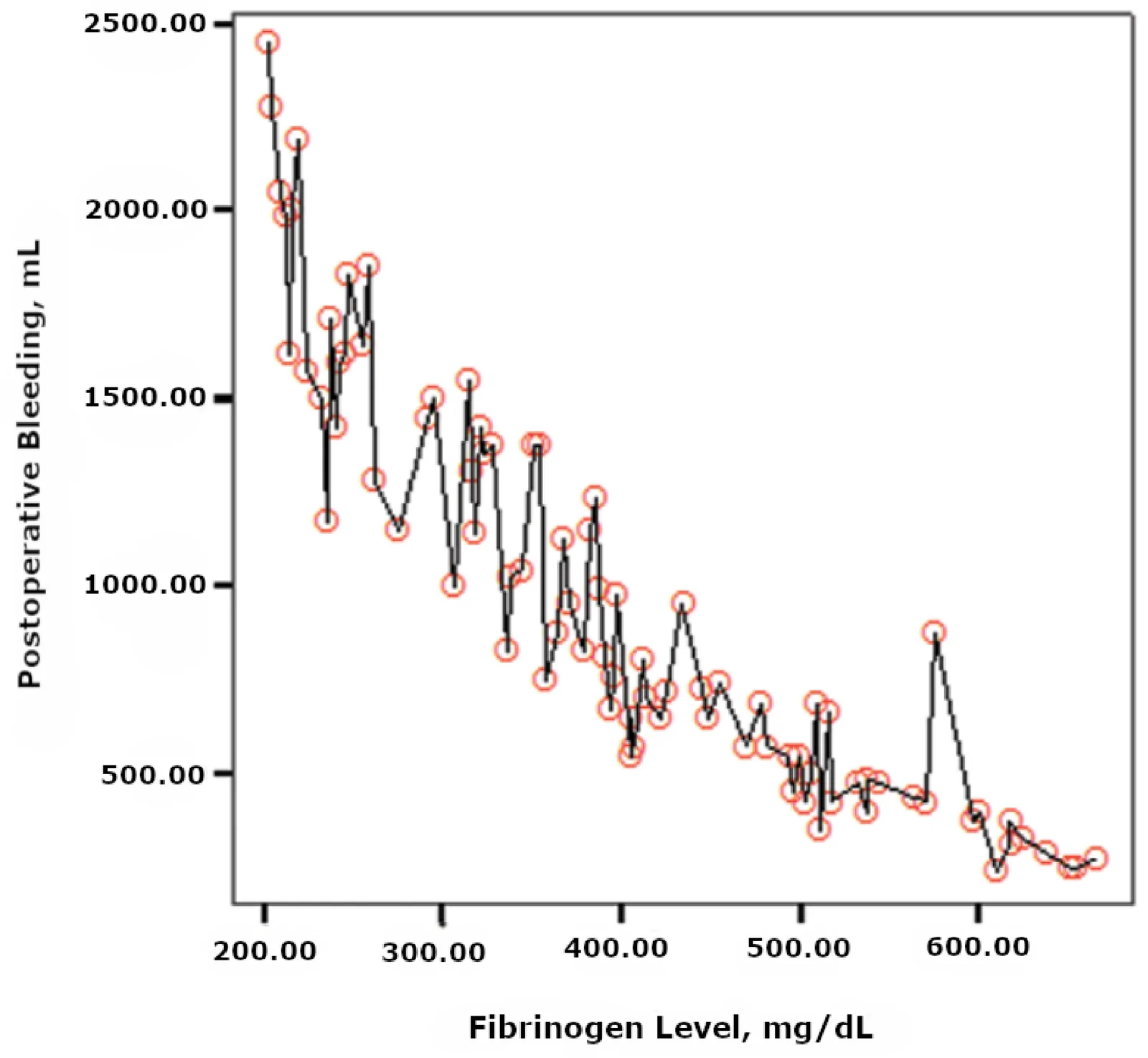
Association between pre-operative fibrinogen levels and the volume of post-operative bleeding via chest tube after open heart surgery (r = −0.897, *p* < 0.001; *n*= 97; (from Ucar et al. [139] published open access under a CC-BY-NC license).

Fibrinogen levels have been shown to be substantially decreased (40–80%) during major cardiovascular surgeries, especially those associated with prolonged periods of cardiopulmonary bypass [1,64,139,140]. There is a strong correlation between pre-operative fibrinogen levels and post-operative bleeding volume (Figure 3) [139]. Another study also found a correlation between pre-operative fibrinogen levels and post-operative bleeding after bypass grafting surgery (r = −0.26, *p* < 0.001) and a correlation of post-operative bleeding with post-operative fibrinogen levels (r = −0.53, *p* < 0.001, *n* = 170) [141].

ROTEM was used to assess the coagulation changes after cardiac surgery [140]. In a patient with normal fibrinogen levels, CT and CFT were slightly increased and α, A10, and MCF were slightly decreased in EXTEM analysis, and A10 and MCF were slightly decreased with FIBTEM.

In a patient with fibrinogen deficiency, CFT was longer and α, A10, and MCF were slightly decreased in EXTEM analysis, and substantial decreases in A10 and MCF were seen with FIBTEM after CPB. Overall, there was a high degree of correlation between fibrinogen levels and A10 and MCF values regardless of the test (EXTEM, INTEM, or FIBTEM; r = 0.69–0.87; *p* < 0.001) [140].

**Figure 4:**
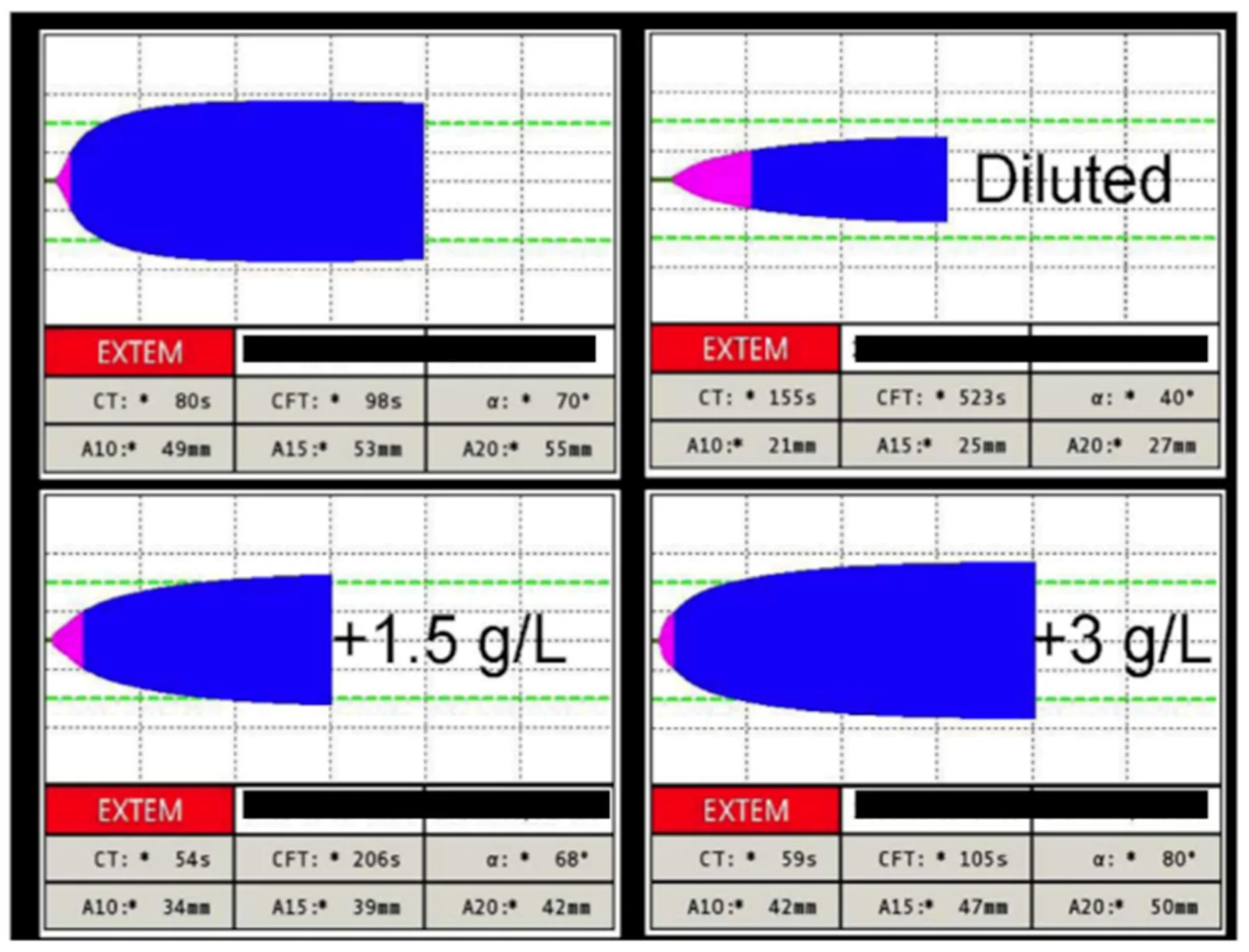
Addition of fibrinogen to diluted whole blood in vitro restores rotational thromboelastometry (ROTEM) parameters. These representative thromboelastometric traces represent how addition of fibrinogen in EXTEM (ROTEM with clotting initiated by tissue factor) improved all thromboelastographic parameters (clotting time (CT), the lag time before clot formation, clot formation time (CFT), α, the angle which reflects the initial rate of clot formation, and A10, firmness amplitude at 10 min (Reprinted with permission from Bolliger et al. 2009; Br J Anaesth 102(6):793–799 [142] with permission from Elsevier).

A study by Bolliger et al. showed that addition of fibrinogen could restore ROTEM parameters when added to diluted whole blood in vitro. This study was designed to mimic hemodilution that occurs when massive hemorrhage is treated with fluids to maintain a normal circulatory volume. Figure 4 shows that dilution of whole blood increased CT and CFT and decreased α and A10. These parameters were partially restored to 1.5 g/L (150 mg/dL) of fibrinogen and fully restored to 3 g/L (300 mg/dL) [142].

A recent survey conducted by the American Society of Anesthesiologists (ASA) found that the most common answer for an adequate fibrinogen level to control bleeding in an actively bleeding patient was 200 mg/dL (35.1%: 314 of 894 respondents). The second most common response was 150 mg/dL (25.8%), and the third most common was “I don’t know” or “I don’t measure fibrinogen levels” (18.9%) [143].

The same survey asked respondents to name the considerations for achieving adequate fibrinogen in a bleeding patient. The most frequent response was efficacy in achieving hemostasis (67.6%). The second and third most frequent responses were cost of treatment (50.7%) and the time required to receive and transfuse the product (35.3%) [143].

## 6. Guidelines for Control of Perioperative Bleeding

**Figure 5:**
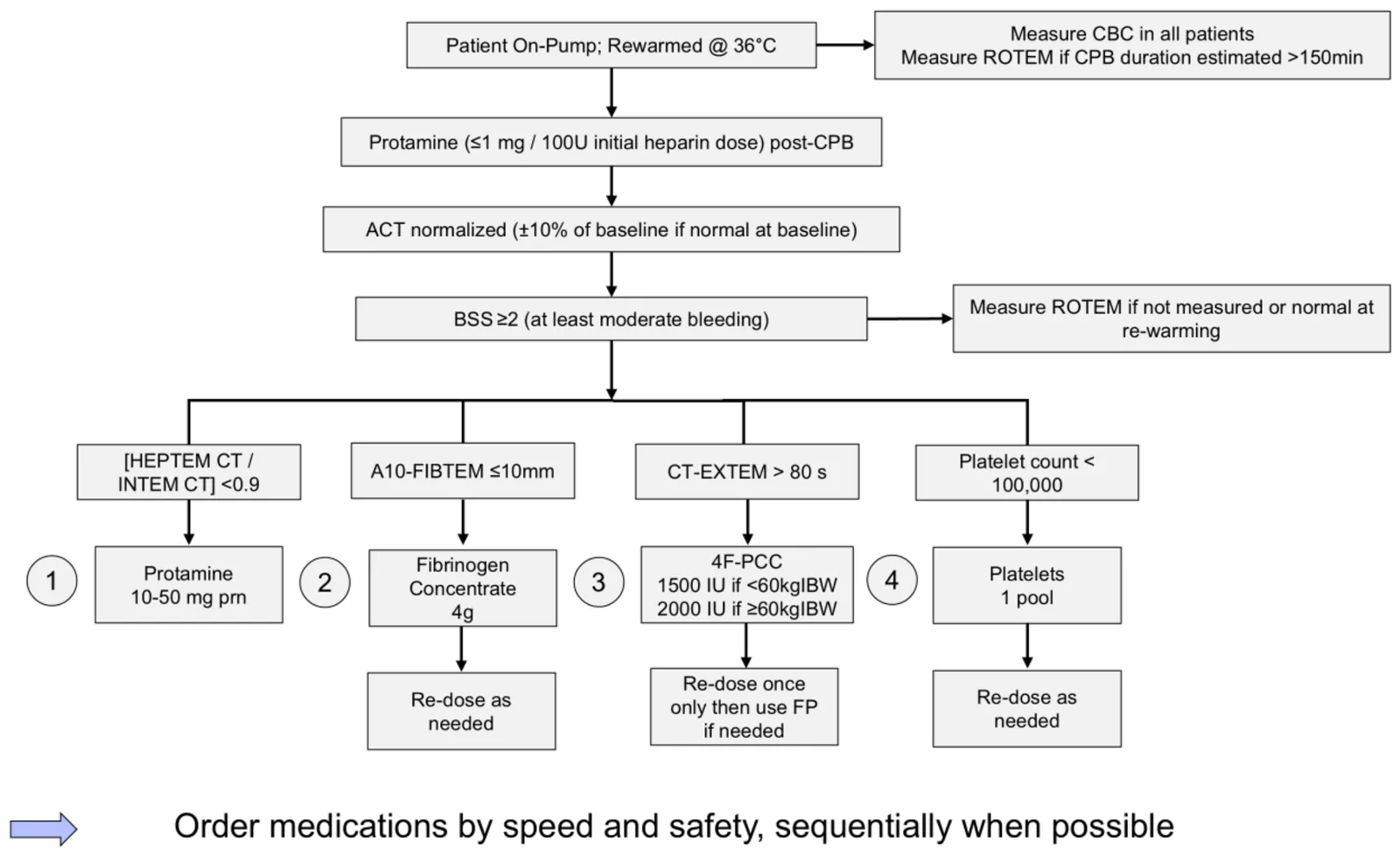
Cardiac surgery blood management algorithm (courtesy of Dr. Jeannie Callum, Department of Laboratory Medicine and Molecular Diagnostics, Sunnybrook Health Sciences Centre, Toronto, Ontario, Canada, adapted from Karkouti et al. [146]). CBC = complete blood count; CPB = cardiopulmonary bypass; ACT = activated clotting time; BSS = bleeding severity score; ROTEM = rotational thromboelastometry; HEPTEM = ROTEM initiated by ellagic acid (INTEM) in the presence of heparinase; FIBTEM = isolated fibrinogen function in ROTEM; EXTEM = ROTEM initiated by tissue factor; 4 F-PCC = four factor (II, VII, IX, X) prothrombin complex concentrate; FP = frozen plasma.

There are several factors to consider in the management of perioperative bleeding; these include the following: (1) There are several sets of guidelines from professional societies [80,84,144]. These guidelines generally include algorithms, and there are local institutional algorithms as well (as shown in Figure 5). (2) The availability of hemostatic testing at the point-of-care is ideal so that real-time measurements can be used to guide care. (3) There are published trigger values to guide treatment, but there is variability in these values, which may affect the utility of trigger values for fibrinogen. (4) There are different agents to treat perioperative bleeding depending on the exact nature of the problem [80,84,144,145].

### 6.1. Current Guidelines and Algorithms

Table 6 shows a section of the algorithm published by Görlinger et al. that deals with excessive bleeding after administration of protamine [147]. This guideline, like most, has trigger values for specified treatments based primarily on retrospective studies. One such study, in 166 patients who had undergone open thoracoabdominal aortic aneurysm repair, examined their A10 FIBTEM on post-surgical ICU admission and the amount of drainage at 24 h [148]. This study found an inverse relationship between A10 FIBTEM and post-operative bleeding at 24 h (r2 = 0.03; *p* = 0.026). Receiver operating characteristic (ROC) analysis showed an area under the curve (AUC) of 0.63 (95% confidence interval (CI) 0.56–0.70; *p* = 0.026). The analysis showed that the best cutoff for predicting severe bleeding was 9 mm. An A10 FIBTEM value of 9 mm showed a negative predictive value for severe bleeding of 92%. The authors concluded that an A10 FIBTEM ≤ 3 mm warranted prompt correction (positive predictive value of 50% for severe bleeding) and that the target A10 FIBTEM should be 9 mm [148]. This A10 FIBTEM (9 mm) correlates with a fibrinogen level just below the lower limit of normal (1.5 g/L (150 mg/dL)) [103]. The validity of these limits is still under discussion.

Retrospective analysis of several coagulation parameters, including fibrinogen levels, to determine associations between these parameters and post-operative blood loss in 2800 cardiac surgery patients was conducted [149]. A negative correlation was found between post-operative bleeding and fibrinogen levels (r2 = 0.043, *p* = 0.001). ROC analysis showed an AUC of 0.647 (95% CI 0.599–0.695). The cutoff level determined in this study was ≥2.2 g/L (220 mg/dL), which had a negative predictive value for severe bleeding of 96.8%. Sensitivity was 54% and specificity was 70% [149].

### 6.2. Trigger Values Versus Testing-Guided Bleeding Management

Trigger values, however, are arbitrary, and their clinical significance is low. In addition, trigger values give no insight into the causality of bleeding. There is no widely accepted evidence-based trigger value for fibrinogen [16]. An uncontrolled prospective study examining the relationship between post-operative fibrinogen levels and post-operative bleeding following cardiothoracic surgery found that low fibrinogen levels were associated with a greater risk of bleeding with an odds ratio of 3.06 for every 1.0 g/L (100 mg/dL) decrease in fibrinogen (95% CI 1.05–8.90). No threshold or trigger level of fibrinogen could be identified [150].

Several recent studies have examined the use of point-of-care testing to affirm the predictive utility of trigger values and the use of viscoelastic and common coagulation tests to guide bleeding management. A recently published study examined the correlation between intraoperative FC administration and increased clot firmness in ROTEM [151]. A 10 mg/kg increase in FC dose was correlated with 1.2 mm increase in A5 FIBTEM. These authors determined a cutoff value for predicting hypofibrinogenemia of A5 FIBTEM at 12 mm with a sensitivity of 76.5% and a specificity of 94.3% [151]. A similar study indicated a threshold of 10 mm with a sensitivity of 70% and specificity of 76% to detect fibrinogen levels < 2.0 g/L (200 mg/dL) [152].

Another recent study found a non-linear relationship between pre-treatment fibrinogen levels and ICU mortality (*p* < 0.001) with a threshold of 1.3 g/L (130 mg/dL) [153]. Post-treatment analysis showed that fibrinogen levels >1.3 g/L (130 mg/dL) were associated with improved survival (*p* < 0.01) and determined a post-treatment target of 2.0–2.5 g/L (200–250 mg/dL). However, their analysis showed several other factors were significantly associated with survival: PT, aPTT, platelet count, white blood cell count, blood pH, bicarbonate, lactate, sodium, potassium, chloride, creatinine, several severity scores, and comorbidities [153].

**Figure 6:**
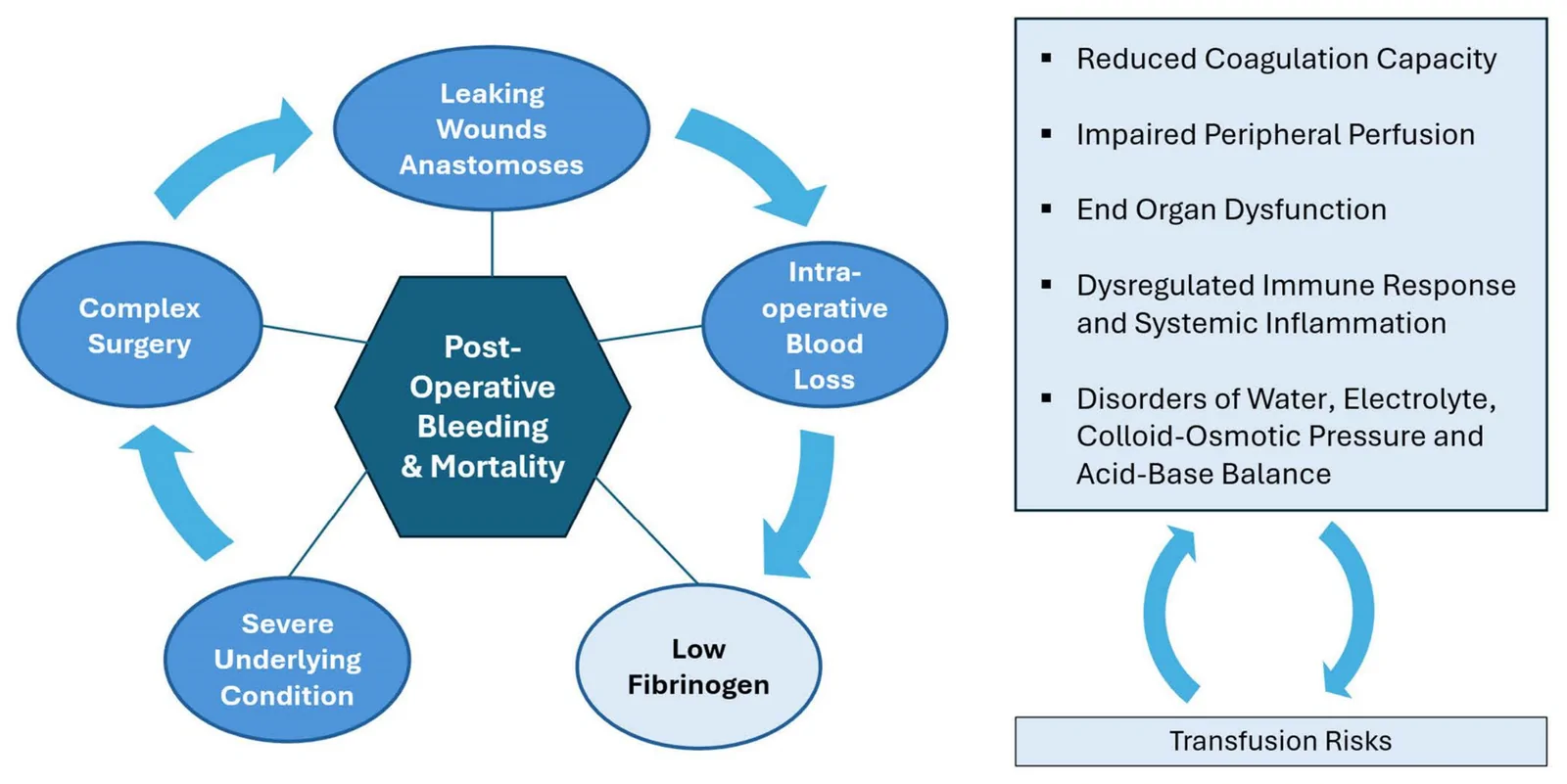
The complex interactions of numerous factors associated with post-operative bleeding and mortality.

**Figure 7:**
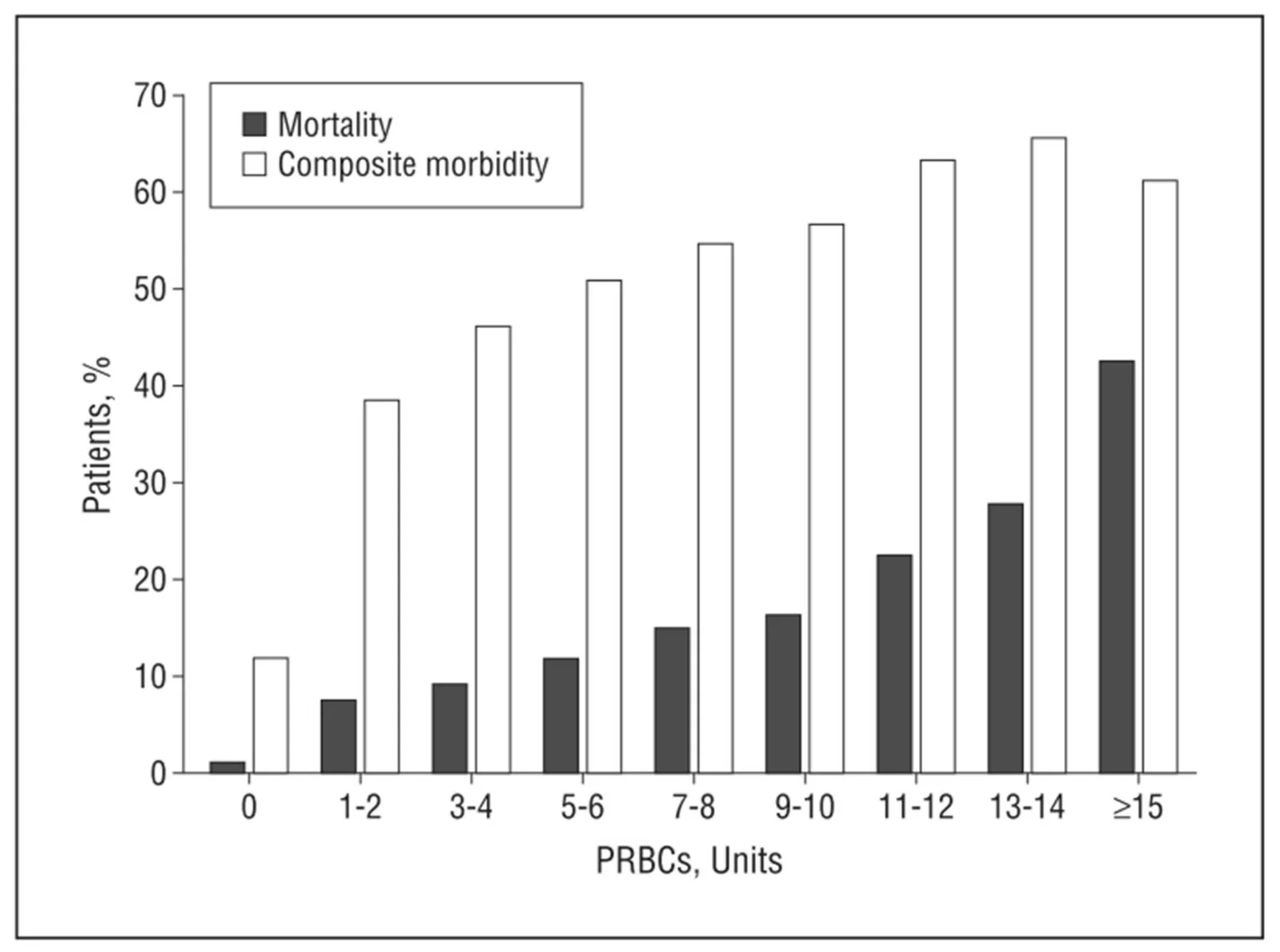
Mortality and composite morbidity rates (unadjusted) by the number of units of packed red blood cells (PRBCs) infused intraoperatively (Reproduced with permission from Ferraris et al. doi:10.1001/archsurg.2011.790. [154] Copyright © 2012 American Medical Association. All rights reserved including those for text and data mining, AI training and similar technologies).

This indicates that although there is an association between low fibrinogen and post-operative bleeding and mortality, other interrelated factors also contribute to these outcomes. These factors also play into the risks associated with intraoperative transfusion (Figure 6). A study of a large population of surgical patients from 173 hospitals in the US (*n* = 941,496) found that a large majority of patients did not receive intraoperative transfusions (94.9%) [154]. Patients who received at least one unit of PRBCs by intraoperative transfusions had higher morbidity and mortality rates than those who did not. An increase in the number of transfusions was associated with a dose-dependent increase in morbidity and mortality (Figure 7) [154].

Similarly, a retrospective study of patients undergoing colorectal cancer surgery found that greater intraoperative blood loss and intraoperative transfusions were associated with poorer outcomes [155]. Short-term outcomes (longer operation times, more complications, and longer hospital stay) were worse in the patients who had higher intraoperative blood loss and intraoperative transfusions compared to those with lower blood loss and no transfusions. These factors were also associated with poorer overall survival [155].

An older study by Hiippala et al. found that when the percentage of circulating blood volume (CBV) lost was considered, fibrinogen was the first of the coagulation components measured to fall below a critical level (1.0 g/L (100 mg/dL) at 142% of CBV). Other components of the coagulation process (platelets, Factors II, V, and VII) require a much higher degree of CBV loss (201–236% of CBV) to reach their critical levels [156].

### 6.3. Other Factors in Guideline Evolution

Modern bleeding management is progressively shifting from fixed laboratory cut-offs toward individualized, goal-directed hemostatic therapy integrating patient characteristics, surgical setting, bleeding severity, viscoelastic testing, and repeated clinical reassessment [157].

Additionally, when considering changes to treatment algorithms or therapeutic guidelines, efficacy is not the only consideration; availability, timely availability, and cost must also be considered. In light of the FIBRES study [65,158], the financial impact of Cryo versus FC was analyzed, leading to changes in treatment algorithms at some hospitals.

A cost-effectiveness analysis was conducted with data from the four Ontario sites from the FIBRES trial [159]. For this subset of patients, efficacy results were similar to those seen in the full FIBRES dataset. The results showed that FC treatment was lower than Cryo. The 7-day median cost in the FC group was CAD $2280 (USD $1698) and CAD $2770 (USD $2063) in the Cryo group. The 28-day cost was CAD $38,180 (USD $28,431) in the FC group and CAD $38,790 (USD $28,886) in the Cryo group. When critically ill patients were excluded (due to extreme variability in costs), FC was found to have a probability of being cost-effective when the willingness to pay limits were set at CAD $0 (86%) and CAD $2000 (USD $1489: 97%) [159].

Based on the results of the FIBRES study, the follow-up cost analysis, and accumulating evidence on the effectiveness of FC, many hospitals in Canada have changed their transfusion choices to favor fibrinogen concentrate over Cryo for post-CPB patients. An example from Kingston Health Sciences Centre and Toronto General Hospital is shown in Figure 5. Viscoelastic testing is generally performed after CPB when patients have separated from CPB and heparin has been reversed with protamine using a stepwise approach based on the coagulation deficiencies identified. If there is bleeding without specific underlying cause of a coagulation disorder, such as pre-operative antithrombotic or antiplatelet therapy, or if there are signs of hypofibrinogenemia, FC is given first. Other factors, e.g., four-factor (II, VII, IX, X) prothrombin complex concentrate (4 F-PCC), are administered if indicated. Allogeneic blood products such as platelets are suggested to be given last if prior measures are unsuccessful.

The rapid availability and ease of access to medications to treat surgical or traumatic bleeding are key. With FC, reconstitution can be performed at the bedside in five minutes or less. This allows for more rapid correction of coagulation problems and control of bleeding, which may help avoid less favorable outcomes.

After publication of the FIBRES study, there was a major change in how perioperative bleeding is treated. Many institutions in Canada no longer use Cryo but have turned to FC instead. In fact, Canadian Blood Services (one of the two major blood suppliers in Canada) reported in 2025 that the use of Cryo in Canada (excluding Quebec) has fallen from 2.3 per 1000 population to 0.1 per 1000 population over the past decade [160].

However, the costs of fibrinogen concentrate compared to cryoprecipitate vary significantly by jurisdiction. Overall, the up-front costs of fibrinogen concentrate may be greater, which on the whole may be offset by decreases in other costs such as reductions in allogeneic blood product use in elective and non-critically ill subgroups. However, the increased up-front costs may drive decision-making at the individual hospital level in health care systems without centralized purchasing of factor concentrates.

## 7. Conclusions

Fibrinogen is a critical factor in clot formation and management of perioperative bleeding. Repletion of fibrinogen in cases of hypofibrinogenemia has been shown to reduce bleeding and the need for allogeneic blood products. Of the products available for repletion, FC has advantages over FP and Cryo in that it is highly purified fibrinogen (i.e., without significant amounts of other coagulation proteins and a reduced risk of viral transmission or immunomodulation) and does not require ABO compatibility. Early recognition of hypofibrinogenemia and targeted replacement therapy can improve surgical outcomes.

Data are accumulating on the relative benefits of FC across many types of surgery. For surgeries where substantial blood loss is expected or occurring, FC can benefit by decreasing blood loss and reducing allogeneic blood product use. The FIBRES study and its follow-up pharmacoeconomic analysis showed that in the conditions of that trial, FC was at least non-inferior to cryoprecipitate while being cost-effective overall and is likely to be more efficacious and even more cost-effective in non-critically ill patients. The efficacy and economic evidence of FC have been associated with decreased use of other fibrinogen replacements (especially Cryo) in Canada.

Regarding the existing guidelines on perioperative bleeding management, the use of algorithms within the guidelines is likely to be beneficial in standardizing care based on clinical evidence, but there is no consensus among the guidelines. A recent scoping review of randomized and observational studies with Cryo and FFP in acquired fibrinogenemia concluded that there was sufficient data to help guide development of new evidence-based guidelines. The authors also noted the need for more comparable and coordinated research [161]. The question of which blood product (FC, FFP, or Cryo) is most commonly used may depend on the individual patient’s clinical circumstances and local product availability and access. Hemostatic testing at the point of care has utility.

However, if point of care testing is not available, the first and most important step in managing severe hemorrhage is usually to administer FC until the bleeding subsides. Active monitoring of the patient’s bleeding status is crucial. At this time, the evidence supporting the use of trigger values is not strong enough to recommend a single value, but most guidelines suggest that at least Claus Fibrinogen values < 1.5 g/L–2 g/L (150–200 mg/dL) [84,85,145,162,163] should be addressed in the context of clinically important bleeding.

Individualized bleeding management must take into consideration the patient’s overall clinical picture. Fibrinogen levels are an important determinant but must be considered in light of each patient’s specific clinical characteristics including co-morbidities, concomitant medications, and the dynamic nature of each clinical scenario.

Additional prospective studies will help clarify the best choice of fibrinogen replacement product and utility of threshold values under different clinical scenarios associated with acquired fibrinogen deficiency. Additional cost-effectiveness studies and wider integration of viscoelastic testing into routine practice and treatment algorithms are also practical steps to potentially improve and personalize treatment of acquired fibrinogen deficiency.

## Figures and Tables

**Table 1:** Preparations for fibrinogen replacement therapy [1,16].

Properties	Fibrinogen Concentrate (FC)	Cryoprecipitate (Cryo)	Fresh Frozen Plasma (FFP)
Active Component(s)	Fibrinogen	FVIII, FXIII, vWF, fibrinogen	All clotting factors and many other proteins
Safety	Viral inactivation Removal of immunomodulatory proteins Minimal contamination with other clotting factors Low thrombogenic potential	No viral inactivation Potential thrombotic risk related to fibronectin, platelet microparticles, and other components including vWF and FVIII	No viral inactivation unless commercially produced Risk of TRALI Risk of TACO
Dosing	Well-defined fibrinogen content Accurate, predictable increase in plasma fibrinogen levels Low volume (approx. 200 mL crystalloid solution per 4 g adult dose)	Variable fibrinogen content due to differences in donors Plasma fibrinogen increase is less predictable Low volume (approx. 250 mL colloid-like solution per 4 g adult dose)	Variable fibrinogen content due to differences in donors Plasma fibrinogen increase is less predictable High volume (approx. 1500–2000 mL colloid-like solution per 4 g adult dose) Modest increase in fibrinogen compared to FC or Cryo
Administration	Reconstituted (~5 min), once reconstituted must be given within 8 h Reduced waste No ABO blood type matching	Thawed (~45 min)–must be given within 4–6 h Potential waste ABO blood matching ideal but may not be required	Thawed (~45 min)–must be given within 24 h if stored between 1 and 6 °C High volume ABO blood type matching required
Storage	In original package, up to 60 months at 2–25 °C	Frozen at –18 °C up to 12 months; potentially up to 36 months if stored at −25 °C or colder	Frozen at –18 °C up to 12 months; potentially up to 36 months if stored at −25 °C or colder

FVIII = Factor VIII; FXIII = Factor XIII: vWF = von Willebrand factor; TRALI = transfusion-related acute lung injury; TACO, transfusion-associated circulatory overload.

**Table 4:** Primary and secondary endpoints from the AdFIrst study (from Rahe-Meyer et al. [45] published open access under a CC-BY license).

	FC	FFP/Cryo	Difference (95% CI)	*p* Value
**Primary Endpoint–Intraoperative Blood Loss (mL)**				
Overall: LSM (95%CI)	1381 (1187–1574)	1160 (1461–18,600)	−279 (−552 to −6)	<0.0001
Spinal Surgery Overall	1613 (1283–1943)	2019 (1665–2374)	−406 (−864 to 52)	0.00075
Spinal Surgery w/o Tumors	1394 (1119–1668)	1861 (1576–2146)	−467 (−821 to −113)	0.00051
Abdominal Surgery	1517 (1131–1703)	1706 (1524–1889)	−190 (−450 to 71)	0.011
**Secondary Endpoints**				
Successful Correction of Fibrinogen: *n pts* (%)				
≤15 min after administration	59 (55)	19 (18)	37 (25–49)	<0.0001
>15 min or ≤90 min	17 (16)	11 (11)	5 (−4 to 14)	
>90 min	11 (10)	16 (15)	−5 (−14 to 4)	
Unsuccessful	20 (19)	58 (56)	−37 (−49 to −25)	
Re-bleed post-surgery to Day 8	0 (0)	5 (5)	−5 (−9 to −1)	0.028
Post-op blood loss First 24 h: mL (95% CI)	206 (234–378)	294 (221–367)	12 (−89 to 114)	0.81
Transfusion requirements: *n pts* (%)				
Any type	79 (74)	72 (69)	5 (−10 to 19)	0.46
RBCs	65 (61)	61 (59)	2 (−11 to 15)	0.76
Length of hospital stay: days (95% CI)	15 (10–19)	13 (10–20)	2 (0–3)	0.43
In-hospital mortality: *n* (%)	0 (0)	0 (0)	0 (0–0)	1.0

Primary efficacy analysis was conducted using the per-protocol patient set (all patients without major protocol deviations), which is shown here. Analysis was also performed in the modified full analysis set (all patients who received at least one dose of trial medication and had at least one post-dose assessment) and yielded similar results. FC = fibrinogen concentrate; FFP = fresh frozen plasma, Cryo = cryoprecipitate; LSM = least squares mean; CI = confidence interval.

**Table 6:** A section of the point-of-care algorithm for coagulation and transfusion management in cardiovascular surgery based on thromboelastometry (ROTEM) and whole blood impedance aggregometry (multiplate) (modified with permission from Görlinger et al. 2013; Curr Opin Anaesthesiol 26(2):230–243 [147]).

ROTEM/Multiplate Results	Recommended Treatment
A10 _EXTEM_ ≤ 40 mm And A10 _FIBTEM_ ≤ 10 mm	FC or Cryo
CT _EXTEM_ > 90 s Or CT _HEPTEM_ > 280 s	PCC or FFP
A10 _EXTEM_ ≤ 40 mm And A10 _FIBTEM_ > 10 mm	Platelets
AUC–ASPI < 200 (or 20 U) AUC–COL < 200 (or 20 U) AUC–ADP < 300 (or 30 U) AUC–TRAP < 500 (or 50 U)	

A10 = firmness amplitude at 10 min; EXTEM = ROTEM initiated by tissue factor; FIBTEM = isolated fibrinogen function in ROTEM; FC = fibrinogen concentrate; CT = clotting time; HEPTEM = ROTEM initiated by ellagic acid (INTEM) in the presence of heparinase; PCC = prothrombin complex concentrate; FFP = fresh frozen plasma; AUC = area under the curve. Multiplate aggregometry: ASPI = with aspirin; COL = with collagen; ADP = with ADP; TRAP = with thrombin receptor-activating peptide-6 (TRAP-6) as the activators.

## Data Availability

Data cited in this article has been previously published and may be available from the cited authors.

## Abbreviations

The following abbreviations are used in this manuscript:

A10	Clot firmness amplitude at 10 min
CFT	Clot formation time
CI	Confidence interval
Cryo	Cryoprecipitate
CT	Clotting time
EXTEM	ROTEM with tissue factor
FVIII	Factor VIII
FXIII	Factor XIII
FC	Fibrinogen concentrate
FFP	Fresh frozen plasma
FIBTEM	ROTEM with cytochalasin D +/− abciximab
FP	Frozen plasma
INTEM	ROTEM with ellagic acid
IQR	Interquartile ratio
LSM	Least squares mean
MCF	Maximum clot firmness
PMP	Pseudomyxoma peritonei
PRBCs	Packed red blood cells
ROTEM	Rotational thromboelastometry
RR	Relative risk
TEG	Thromboelastography
TRALI	Transfusion-related acute lung injury
TACO	Transfusion-associated circulatory overload
vWF	von Willebrand factor
